# Supplementary figures and images for: Integrated cross-organ transcriptomic analysis uncovers conserved gene signatures predictive of allograft rejection
Source: PLoS One. 2026 Apr 30;21(4):e0348135. doi: 10.1371/journal.pone.0348135 (PMC13132223; doi:10.1371/journal.pone.0348135)

## Liver

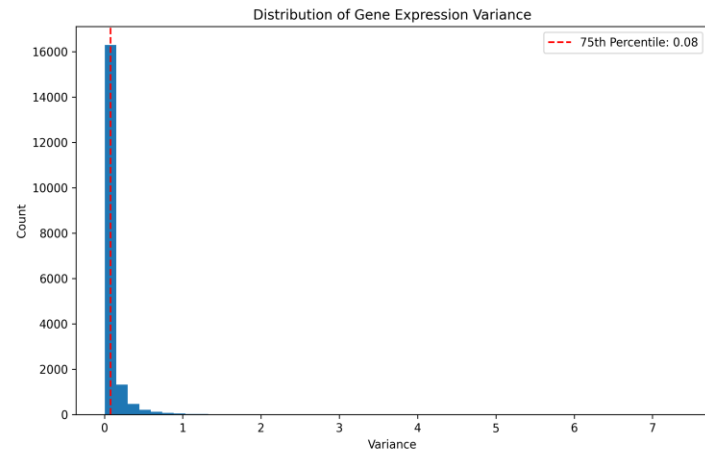

## Kidney

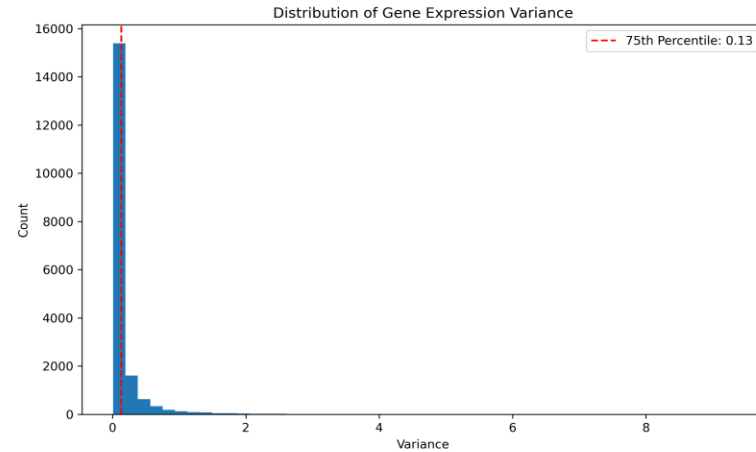

## Heart

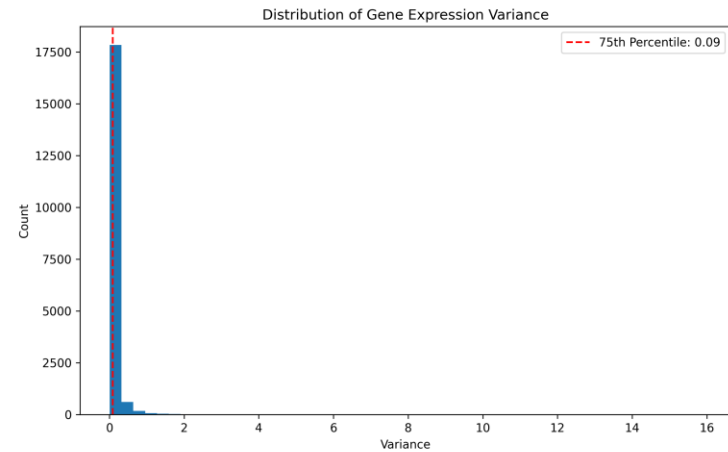

Supplement: S1 Fig — Histograms of gene-wise variance for liver, kidney, and heart datasets illustrating a right-skewed distribution, motivating retention of the top 25% most variable genes. (PDF) [file pone.0348135.s001.pdf]

Nodes vs Correlation Threshold

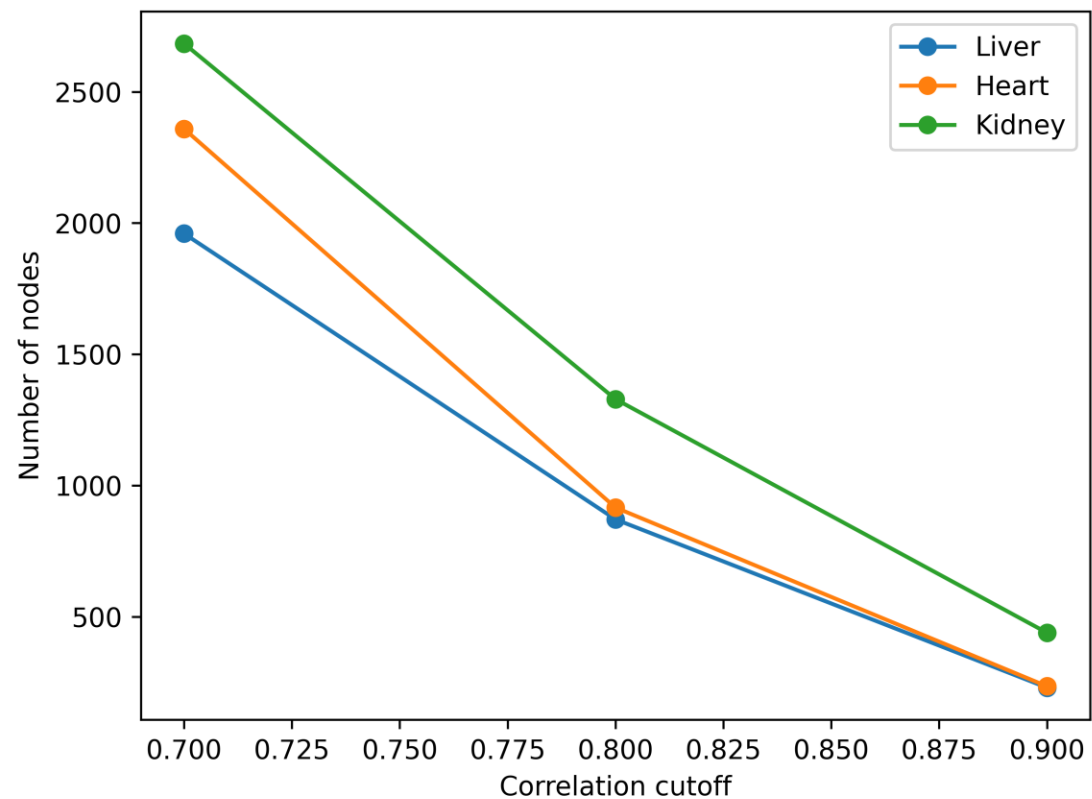

Edges vs Correlation Threshold

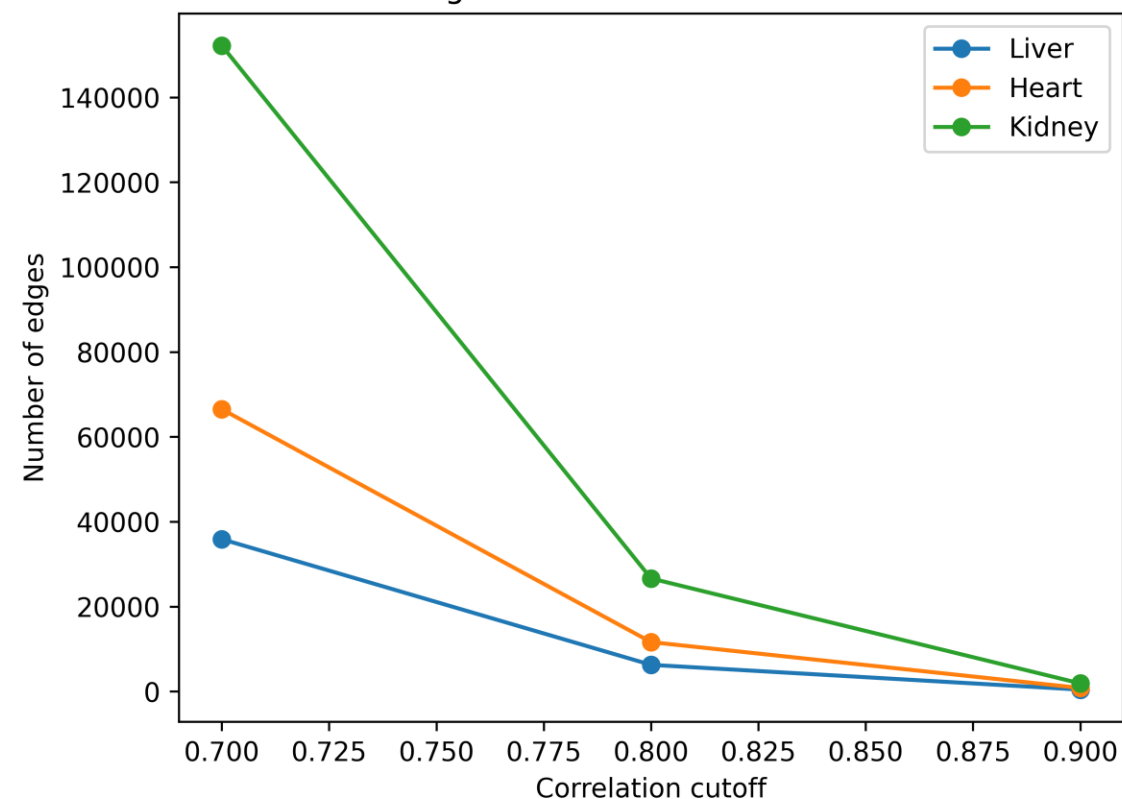

Supplement: S2 Fig — Number of nodes and edges retained across correlation thresholds (|r| = 0.7–0.9) for liver, kidney, and heart co-expression networks. Lower thresholds produce dense networks, while higher thresholds fragment the network, supporting the choice of |r| > 0.8. (PDF) [file pone.0348135.s002.pdf]

Number of Modules vs Leiden Resolution

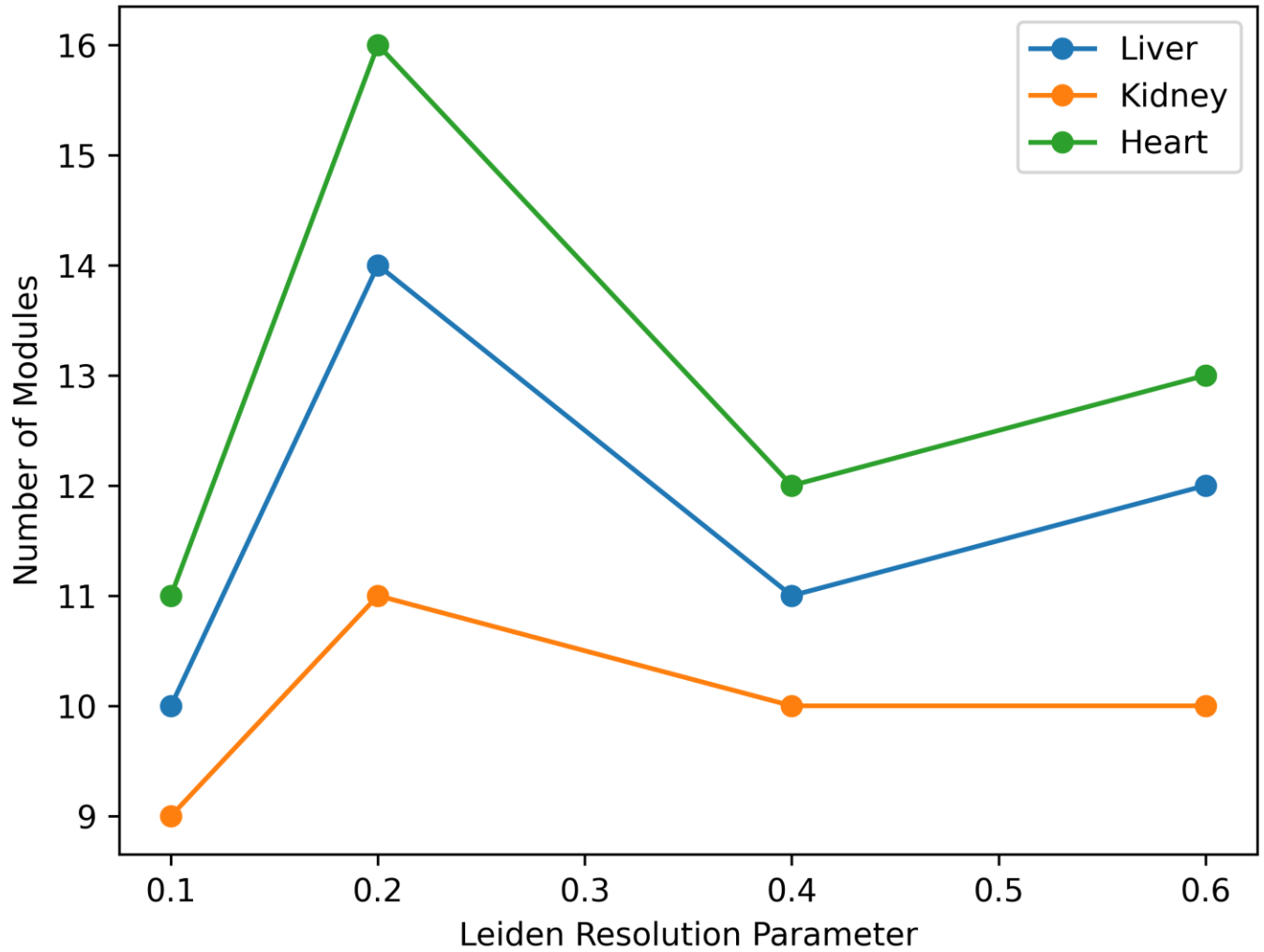

Supplement: S3 Fig — Number of modules detected across Leiden resolution parameters (0.1–0.6) for liver, kidney, and heart networks. Resolution = 0.2 produced the most number of modules with biologically interpretable sizes. (PDF) [file pone.0348135.s003.pdf]

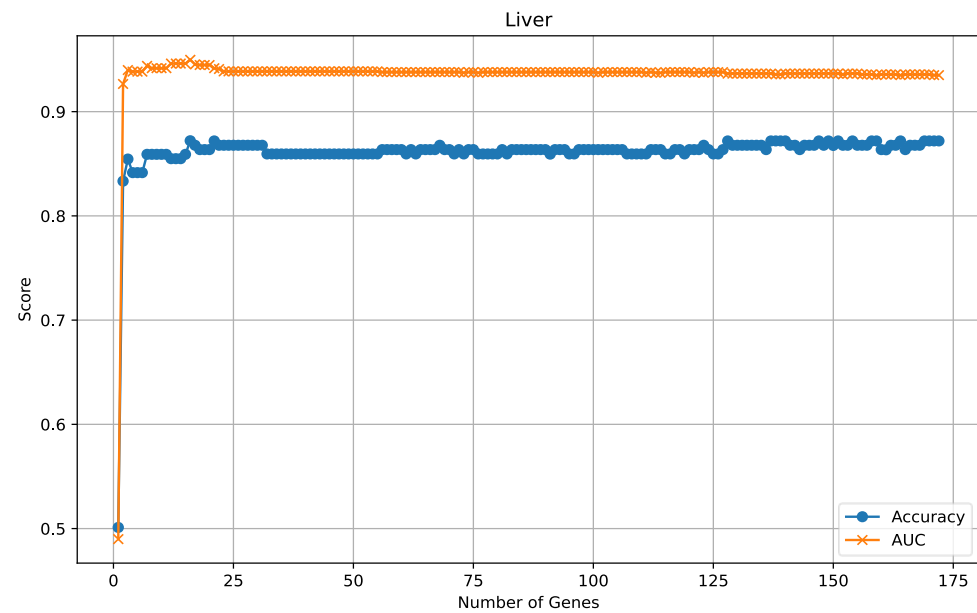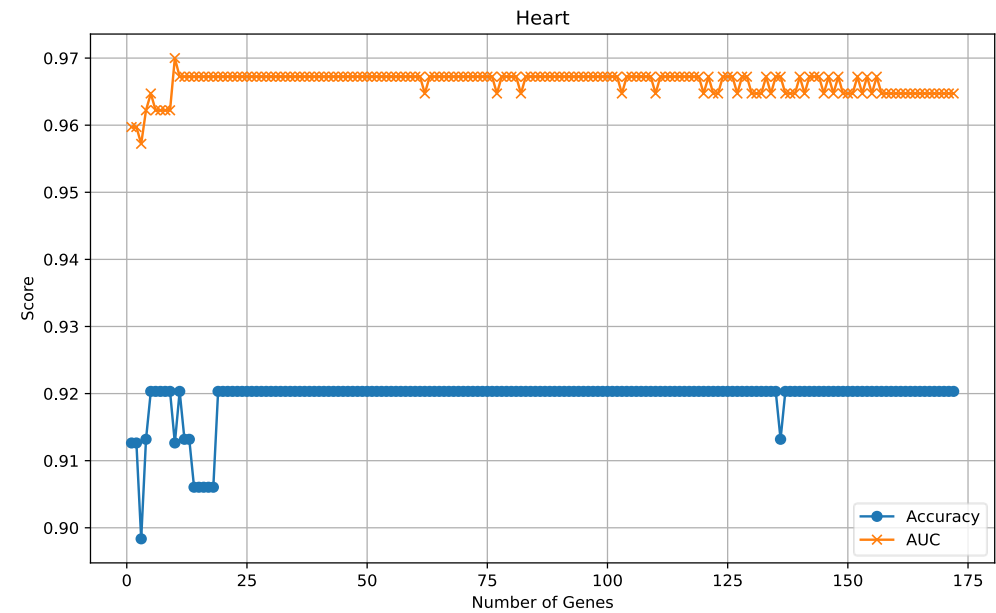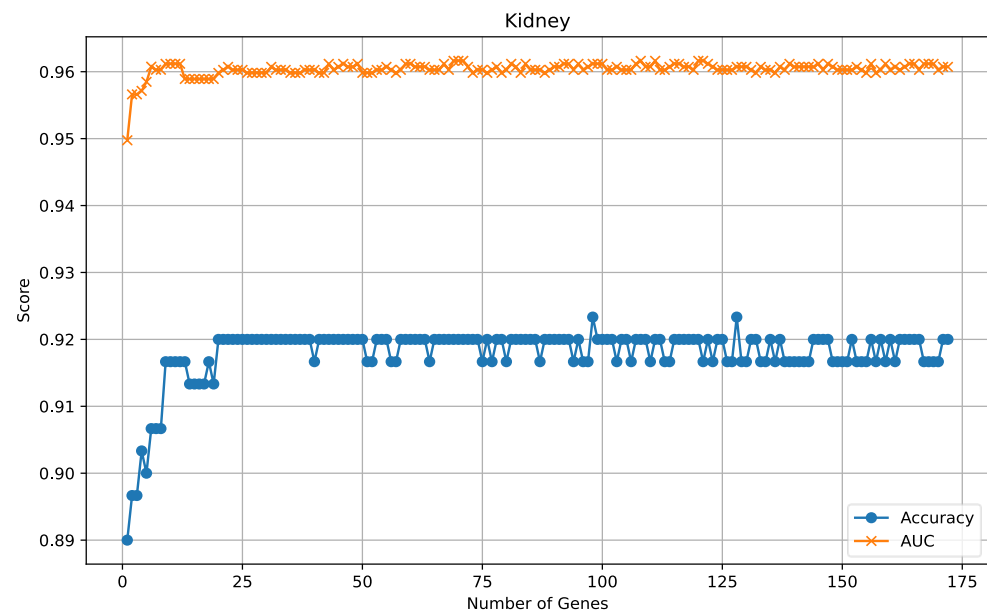

Supplement: S4 Fig — Classifier accuracy and AUC evaluated as a function of feature number for liver, kidney, and heart datasets. Performance plateaued beyond ~20 genes, supporting selection of a compact 20-gene biomarker panel. (PDF) [file pone.0348135.s004.pdf]
